# Epithelial Barrier Function and Altered Cell Signaling Pathways in the Esophageal Epithelium of Achalasia Patients

**DOI:** 10.5152/tjg.2025.25031

**Published:** 2025-10-10

**Authors:** Sezgi Kipcak, Pelin Ergun, Nur Selvi Gunel, Serhat Bor

**Affiliations:** 1 Department of Medical Biology, Ege University Medical School, Izmir, Türkiye; 2 Department of Otolaryngology and Communication Sciences, Medical College of Wisconsin, Wisconsin, USA; 3 Division of Gastroenterology, Ege University Medical School, Izmir, Türkiye

**Keywords:** Achalasia, cell signaling pathway, epithelial barrier function, permeability, tissue resistance

## Abstract

**Background/Aims:**

Idiopathic achalasia is a rare esophageal motility disorder of unknown etiology. Although its neuromuscular aspects are well described, little is known about the role of the esophageal epithelium. This study aimed to evaluate the activation status of key cell signaling pathways and assess esophageal epithelial barrier function in achalasia patients.

**Materials and Methods:**

Biopsy samples from 37 achalasia patients and 15 healthy volunteers (HVs) were analyzed. Tissue resistance and permeability were measured using a mini-Ussing chamber system. Gene expression related to epithelial integrity and signaling was assessed via quantitative reverse transcription polymerase chain reaction, and corresponding protein levels were evaluated using enzyme-linked immunosorbent assay (ELISA) and multiplex ELISA.

**Results:**

No significant differences were observed in epithelial resistance (achalasia: 187.3 ± 25.6 Ω vs. HVs: 166.8 ± 20.1 Ω, *P* = .18) or permeability (achalasia: 35.76 ± 5.4 pmol vs. HVs: 36.9 ± 4.7 pmol, *P* = .67) between the 2 groups. Thirty-two genes involved in key signaling pathways were found to be significantly deregulated (*P* < .05), and 6 key signaling proteins (Akt (Ser473), c-Jun (Ser63), Erk1/2 (Th202/Tyr204), Thr185/Tyr187), IκB-α (Ser32/Ser36), MEK1 (Ser217/Ser221), mTOR (Ser2448)) were downregulated at the protein level (*P* < .05).

**Conclusion:**

The findings reveal that major signaling pathways, including MAPK, PI3K/AKT/mTOR, and JAK/STAT, are significantly suppressed in the esophageal epithelium of achalasia patients, despite preserved epithelial barrier integrity. These molecular alterations may represent a previously unrecognized component of achalasia pathogenesis. Furthermore, the preserved barrier function suggests that endoscopic therapies such as peroral endoscopic myotomy may not exacerbate reflux-related epithelial injury in these patients.

**Main Points**

- Esophageal epithelial barrier function remains intact in achalasia patients, with tissue resistance and permeability similar to those of healthy volunteers, indicating no predisposition to reflux-related damage post-treatment.
- Key cell signaling pathways, including MAPK, PI3K/AKT/mTOR, and JAK/STAT, show reduced activity in achalasia, suggesting impaired molecular signaling in the esophageal epithelium.
- Thirty-two genes related to cell signaling were deregulated, and 6 proteins involved in these pathways were significantly lower in achalasia patients compared to healthy controls.
- The study provides novel insights into the molecular and electrophysiological properties of the esophageal epithelium in achalasia, potentially guiding future research on disease mechanisms and treatment outcomes.

## Introduction

Idiopathic achalasia is a rare disease with an incidence of 1-10/100 000 and occurs with equal frequency in men and women.^1^ The disease is characterized by the absence of esophageal peristalsis and impaired relaxation of the lower esophageal sphincter (LES).^2^ The diagnosis is usually delayed 3-4 years because of its low prevalence and symptoms similar to those of gastroesophageal reflux disease (GERD).^3^ Accordingly, there is often a long time between the onset of symptoms and diagnosis and treatment.^4^

Histopathological findings in idiopathic achalasia reveal the loss of ganglion cells in the myenteric plexus of the esophagus and LES, often accompanied by inflammation and collagen deposition.^5^ Although its precise etiology remains unclear, it is widely accepted that a combination of autoimmune mechanisms, viral triggers, and genetic predisposition contributes to disease development. For instance, single nucleotide polymorphisms in the nitric oxide synthase 1 gene and the interleukin-23 receptor gene expressed by Th17 cells have been associated with achalasia.^6^^,^^7^ However, studies on the genetic changes that cause the onset of achalasia and occur in the disease are very limited.

Although the primary targets of the disease are the esophageal muscle layers and enteric neurons, it is important to investigate the role of the esophageal epithelium as well. Current literature on potential epithelial alterations in achalasia is scarce. Due to the failure of LES relaxation, retained food and secretions can remain in the esophagus for prolonged periods.^2^ This may affect the esophageal epithelial barrier through mechanical irritation or lactic acidosis resulting from stasis-related fermentation.

The primary function of the esophageal epithelial barrier is to protect the esophagus from harmful intraluminal contents.^8^ This barrier is maintained by the apical junctional complex (AJC), which connects adjacent epithelial cells and regulates both paracellular permeability and intercellular signaling.^9^ Evaluation of the expression of molecules associated with the AJC and electrophysiological measurement of the transepithelial resistance (TER) of epithelial tissue allows determination of epithelial barrier function properties.

The main idea underlying all therapeutic approaches is related to the opening of the LES with the disruption of muscles. Balloon dilation and especially peroral endoscopic myotomy (POEM) are related to severe GERD in some cases. Knowledge of the barrier properties of the esophageal epithelium help in further evaluating the mechanisms responsible for GERD.

Therapeutic strategies in achalasia aim to relieve functional obstruction at the LES, primarily through endoscopic or surgical myotomy. Procedures such as balloon dilation and POEM are effective in symptom control but are associated with a high incidence of post-treatment GERD.^10^^,^^11^ Assessing the epithelial barrier function in these patients may help clarify whether epithelial vulnerability contributes to reflux-associated injury, particularly after LES-disrupting interventions.

The aim of this study was 2-fold: (1) to determine the activation status of important cell signaling pathways in achalasia and (2) to evaluate the esophageal epithelial barrier function in achalasia using electrophysiological and molecular methods. It is especially important to examine the epithelial barrier function characteristics in achalasia and to determine the predisposition of the epithelium to GERD, which is common after treatment.

## Materials and Methods

### Ethical Approval

All procedures performed in this study involving human participants were conducted in accordance with the ethical standards of the Ege University and with the Helsinki Declaration and its later amendments or comparable ethical standards. Ethics committee approval of the study was obtained from the Ege University Clinical Research Local Ethics Committee (Approval Number: 8-10.1T/27, October 17, 2018 and Approval Number: 18-2.1/36, February 10, 2018). Written informed consent was obtained from all individual participants included in the study.

### Study Population

Thirty-seven patients whose barium esophageal radiography, high-resolution motility (HRM) test, and upper gastrointestinal endoscopy findings were compatible with achalasia and 15 healthy volunteers (HVs) were included in the study. The HVs had normal upper gastrointestinal endoscopy, intraesophageal 24-hour MII-pH, and HRM results; in addition, they had no history of upper GI disease or surgery. Subtypes of achalasia patients were determined by HRM (MMS – Laborie, the Netherlands) according to the Chicago-IV classification.^12^ Data from 11 type I, 21 type II, and 5 type III achalasia patients were used. All subjects were newly diagnosed and had not received prior treatment. Baseline characteristics are given in Table 1.

The exclusion criteria for the study subjects were Barrett’s esophagus, primary esophageal motility disorders (except achalasia), upper GI surgery, and other disorders that may affect the results, such as cancer, severe coronary artery disease, chronic obstructive pulmonary disease, and uncontrolled diabetes mellitus.

Sample size was determined based on the feasibility of biopsy collection and prior literature on Ussing chamber studies. Although a formal power analysis was not performed, the sample size was deemed appropriate to detect meaningful molecular and electrophysiological differences between groups.

### Upper Gastrointestinal Endoscopy and Biopsy Collection

All endoscopic procedures in the study were performed by the same endoscopist (S.B.) with the assistance of a trained technician. After routine upper gastrointestinal endoscopies were completed, 6 esophageal biopsies were obtained from each subject, 3-5 cm above the Z-line, a region selected to avoid gastric contamination and to represent non-cardial squamous epithelium (radial jaw 4, opening diameter of 2.8 mm; Boston Scientific, USA).

Three biopsy materials were immediately placed in ice-cold preoxygenated Ringer’s solution for use in the in vitro mini-Ussing chamber system measurements. One biopsy was preserved in RNA-stabilizing reagent for use in gene expression analysis and stored at −80°C until total RNA isolation. Two biopsy materials were snap-frozen in liquid nitrogen and stored at −80°C for protein extraction.

### In Vitro Mini-Ussing Chamber Studies

The chambers were filled with Ringer’s solution carbonized with O_2_/CO_2_ (95/5%) at 37°C to provide an incubation medium for the tissue. After 30 minutes of calibration, 3e biopsy materials were mounted into 3 mL Ussing chambers (Scientific Instruments, Simmerath, Germany) modified with a 0.017 cm^2^ adapter under a light microscope.

After all the tissues were placed in the system, the measurement was started, and the electrophysiological properties of the tissues were recorded for 150 minutes. The experiments were performed under open-circuit conditions. Tissues with baseline transepithelial electrical resistance (TEER) values <50 Ω·cm^2^ were excluded.

Thirty minutes after the electrophysiological measurements of the tissues in mini-Ussing chambers, fluorescent dye was added to the apical sides of the tissues (100 mg/mL) (Fluorescein, 376 Da, Sigma Aldrich, St. Louis, MO, USA), and samples were taken from the basolateral side at half-hour intervals. At the end of the experiment, fluorometric measurements of all of the samples were taken in a FLUOstar OPTIMA (BMG Labtech, Ortenberg, Germany) device, and permeability results were obtained.

### Gene Expression Studies

Total RNA was isolated from biopsy materials using an Aurum™ Total RNA Mini Kit (Bio-Rad Laboratories, Inc., Hercules, CA). A Bioprep-6 Homogenizer (Hangzhou Allsheng Instruments Co., Ltd) was used for the homogenization of biopsy tissues. The cDNA was synthesized from the isolated total RNA using an iScript cDNA Synthesis Kit (Bio-Rad Laboratories, Inc., Hercules, CA). SYBR Green-based quantitative reverse transcription polymerase chain reaction (qRT-PCRs) samples were prepared using an iTaq Universal SYBR® Green Supermix kit (Bio-Rad Laboratories, Inc., Hercules, CA).

Primers for 6 molecules associated with epithelial barrier function (E-cadherin, *CDH1*; Claudin 1, *CLDN1*; Claudin 4, *CLDN4*; Zonula occludens 1, *ZO-1*; Zonula occludens 2, *ZO-2*; and Occludin, *OCLN)* were obtained from GeneCopoeia, and panels containing genes associated with cell signaling (Human JAK/STAT Signaling Primer Library ve Human NFKappaB Primer Library) were obtained from Real Time Primers, LLC. All qRT-PCR studies were performed with a LightCycler® 480 (Roche Diagnostics Inc., Basel, CH) instrument.

The 2^−ΔΔCt^ method was used for quantitative analysis of genes. As a result of pairwise comparisons between the groups, gene values with *P* values less than .05 and those with fold changes of ±2 or more were evaluated.

The String Consortium 2020 database was used for pathway analysis of genes showing statistically significant expression changes. A 0.400 medium CI was chosen as the minimum required interaction score, and pathway analysis data from the Kyoto Encyclopedia of Genes and Genomes was used.

### Protein Expression Studies

Proteins were extracted using the Universal Protein Extraction Reagent (BioTeke, China) and quantified via the Lowry method. Protein levels of CDH1, CLDN1, CLDN4, ZO-1, ZO-2, and OCLN were quantified using ELISA kits (Sun Red Biotechnology), and signal intensities were read using a Varioskan™ Flash reader (Thermo Scientific).

Levels of 7 proteins related to cell signaling were analyzed using the Multiplex ELISA method, and a Bio-Plex Pro Cell Signaling Phospho 7-plex panel (Akt (Ser473), c-Jun (Ser63), Erk1/2 (Th202/Tyr204), Thr185/Tyr187, IκB-α (Ser32/Ser36), MEK1 (Ser217/Ser221), mTOR (Ser2448), and Stat3 (Tyr705)) (Bio-Rad Laboratories, Inc., Hercules, CA) was used.

### Statistical Analysis

Statistical analysis was conducted using IBM® SPSS® Statistics 25.0 (IBM SPSS Corp.; Armonk, NY, USA). Normality was assessed by the Shapiro–Wilk test. Between-group comparisons were made using independent *t*-tests for normally distributed variables and Mann–Whitney *U* or Kruskal–Wallis tests for nonparametric data. One-way ANOVA was used for protein comparisons among subtypes. A *P*-value < .05 was considered statistically significant.

## Results

### Determination of Epithelial Barrier Function Properties Via Mini-Ussing Chamber Studies

**Figure 1.**
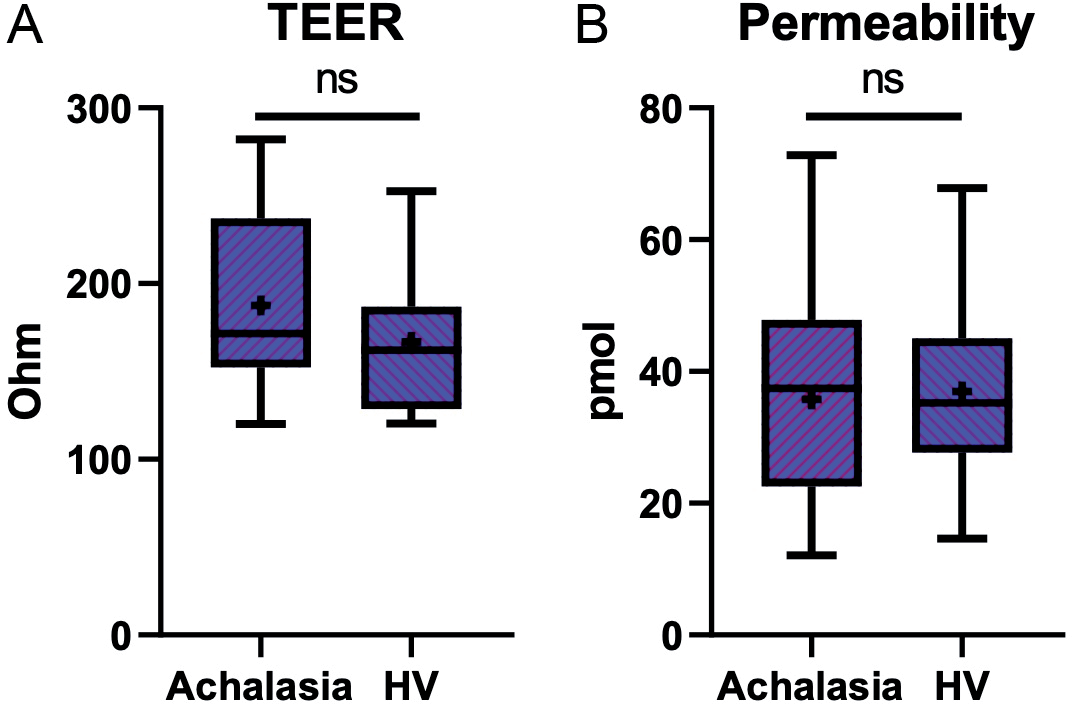
Transepithelial resistance and permeability measurements of esophageal biopsies from patients with achalasia and healthy volunteers. (A) Transepithelial electrical resistance (TEER) was slightly higher in achalasia patients compared to healthy volunteers (HVs), but the difference was not statistically significant (*P* = .06). (B) Epithelial permeability, assessed by fluorescein transport, showed no significant difference between the 2 groups (*P* = .76).

It was determined that the achalasia group has TEER characteristics similar to those of HVs. The TEER values (187.3 Ω ± 15.15) in the achalasia patients were numerically higher than those in the HVs (166.8 Ω ± 13.7), without reaching a significant difference (*P* = .06) (Figure 1A).

Similarly, there were no significant differences in epithelial permeability as assessed by fluorescein diffusion between groups (*P* =  .76). The TEER and permeability results were mutually consistent, suggesting preserved epithelial barrier integrity in achalasia patients (Figure 1B).

### Expression of Epithelial Barrier Function-Related Genes and Proteins

The expression levels of 6 genes (*CDH1*,* CLDN1*,*CLDN4*,* ZO-1*,* ZO-2*,and *OCLN*) related to epithelial barrier function were significantly higher in achalasia patients than in HVs (Table 2). *ZO-2* was the gene with the highest expression increase (a 5.9-fold change), while *OCLN* was the gene with the lowest expression difference (a 2.97-fold change).

At the protein level, among the same 6 targets, only ZO-2 showed significantly higher expression in the achalasia group (*P* < .05), while the others did not differ significantly (Table 3). This discrepancy between gene and protein levels may be attributed to post-transcriptional or post-translational regulatory mechanisms.

### Expression of Cell Signaling Genes and Proteins

Of the 180 analyzed cell signaling-related genes, 32 were significantly deregulated between the achalasia group and HVs (*P* < .05) (Table 4). The *BCL2L1*,* IFNA1*,and *IL10* genes showed an over 20-fold increase in expression. The expression levels of the *STAT1*,* H-RAS*,and *KRAS* genes were decreased 5.72-fold, 4.01-fold, and 3.77-fold, respectively.

**Figure 2.**
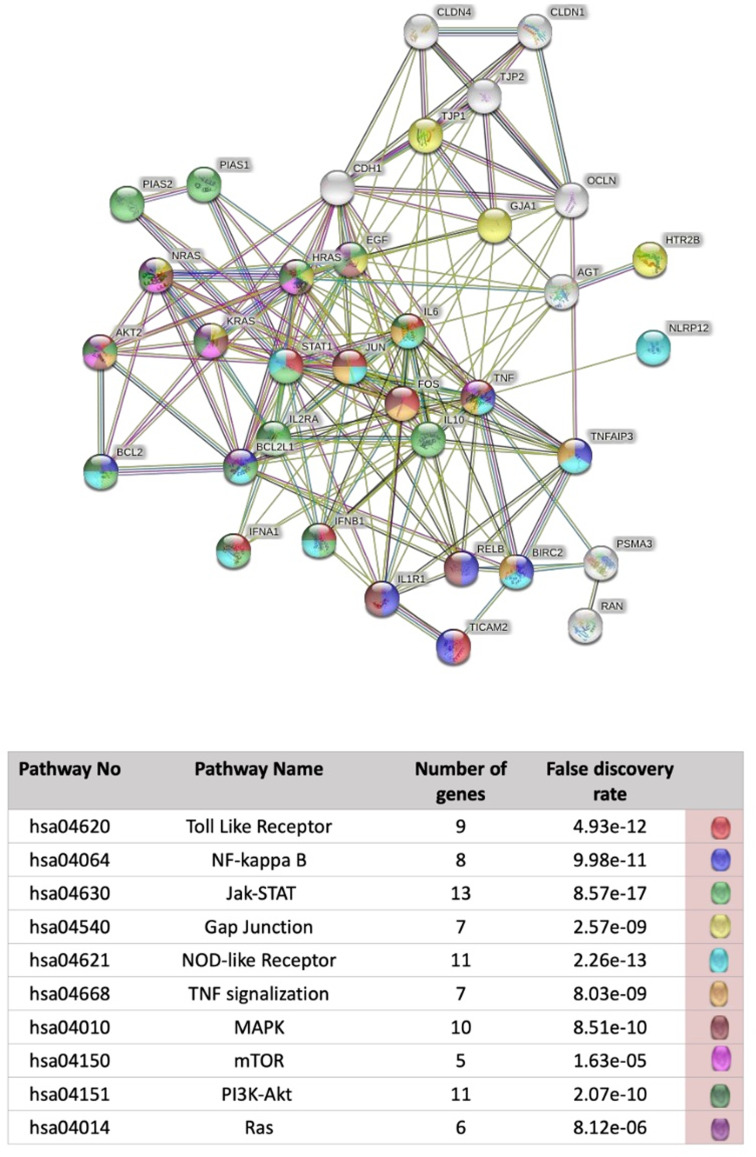
Gene interaction network and pathway enrichment analysis of differentially expressed genes in achalasia patients. The figure presents a STRING-based interaction map of genes with significantly altered expression in achalasia compared to healthy volunteers (HVs). The lower panel lists the top 10 enriched pathways based on KEGG annotation. The analysis was conducted using a medium confidence threshold (interaction score ≥ 0.400), and results are ranked by false discovery rate (FDR).

The results of pathway analysis of genes with expression differences in the achalasia group compared to the HV group, created with the STRING program, are shown in Figure 2. Ten signaling pathways associated with genes with expression changes were determined.

Protein levels of 7 phosphorylated molecules involved in cell signaling (Akt (Ser473), c-Jun (Ser63), Erk1/2 (Th202/Tyr204), Thr185/Tyr187), IκB-α (Ser32/Ser36), MEK1 (Ser217/Ser221), mTOR (Ser2448), and Stat3 (Tyr705)) were significantly lower in achalasia patients than in HVs, except for STAT3 (Table 5).

Collectively, these findings confirm that key signaling pathways—including MAPK, PI3K/AKT/mTOR, and JAK/STAT—are markedly downregulated at both the gene and protein levels in the esophageal epithelium of achalasia group.

## Discussion

The esophageal epithelium, which is indirectly affected by the loss of peristalsis in achalasia, has been shown to exhibit distinct molecular and electrophysiological features compared to those of HVs.

One of the aims of the study was to determine the activation states of molecular signaling pathways in the esophageal epithelium in patients with achalasia. According to the results, important molecular data were obtained showing activation of the MAPK, NF-kB, PI3K-AKT, and JAK-STAT signaling pathways.

Ras/RAF/MEK/ERK signaling is a central pathway that regulates cellular proliferation, differentiation, and survival.^13^ Epidermal Growth Factor (EGF) binds to the EGF receptor and initiates the MAPK cascade.^14^ The *EGF* gene levels in achalasia patients were found to be lower than those in HVs. Additionally, the gene expression levels of* H-ras* and *N-ras*,^15^ which are molecules responsible for activating RAF in the MAPK pathway, were decreased in the achalasia group compared to HVs. The gene expression levels of *MEK1 (MAP2K1)*, which is phosphorylated by RAF, and *ERK2 (MAPK1)*, which is phosphorylated by MEK1,^16^ were lower in achalasia group than in HVs. Additionally, the protein expression levels of the phosphorylated forms of MEK1 and ERK1/2 proteins were investigated, and MEK1 and ERK1/2 protein levels were found to be significantly lower in achalasia groups than in HVs. The entry of the ERK1/2 protein into the nucleus via active transport is dependent on RAN.^15^ In this study, *RAN* gene levels in achalasia group were lower than those in HVs. ERK1/2 protein acts as a transcription factor in c-Jun expression by binding to the TRE region of the *c-Jun* gene promoter.^17^ It was found that c-Jun protein was expressed at a low level in achalasia compared to HVs. *FOS* gene expression is induced by the MAPK pathway;^18^ *FOS* gene expression levels were also lower in the achalasia group than in the HV group. By examining the expression levels of molecules related to the MAPK signaling pathway, it was determined that the activity of the MAPK pathway was low in achalasia patients. The observed downregulation of the MAPK signaling pathway may have functional implications beyond epithelial dynamics. Given its known role in smooth muscle contraction, cellular migration, and neuromuscular regulation,^19^^,^^20^ reduced MAPK activity could potentially contribute to the impaired relaxation of the LES and disordered motility characteristic of achalasia. Although the direct mechanistic links remain to be elucidated, these alterations may affect the function of esophageal smooth muscle cells and enteric neurons. To the best ofr knowledge, no previous data have been reported concerning the activation of the MAPK signaling pathway in achalasia, and the study might be pioneering.

NFκB is a family of transcription factors that play a central role in inflammatory response coordination. These transcription factors are involved in cellular differentiation, proliferation, and survival.^21^ The expression levels of 8 genes that play a role in NFκB signaling pathway activation were found to be high in the achalasia group. However, the protein level of the phosphorylated form of IκB-α was investigated, and the protein level in the achalasia group was significantly lower than that in the HV group. The inconsistency between the gene and protein results suggests that examining the phosphorylated form of IκB-α is not sufficient to interpret pathway activation. The protein expression levels of both the nonphosphorylated form of IκB-α and other important molecules in the pathway need to be examined. Additionally, it is known that transcription of the BCL2 molecule occurs via the NF-κB pathway.^22^ In the study, *BCL2* and *BCL2L1* gene expression levels were significantly higher in achalasia group. This may provide evidence that the NF-κB pathway is more active in achalasia patients than in HVs. NF-κB has been implicated in GERD.^23^ It is possible that NF-κB activation in achalasia is related to esophageal inflammation caused by food retention and potential reflux.

The PI3K-AKT pathway is an intracellular signal transduction pathway that promotes metabolism, proliferation, cell survival, growth, and angiogenesis in response to extracellular signals.^24^ The gene expression levels of* EGF*, which activates the PI3K-AKT pathway, and the PI3K catalytic subunit *PIK3CB* were lower in achalasia group than in HVs. Phosphorylated AKT phosphorylates the mTOR protein via Rheb.^25^ In this study, phosphorylated mTOR levels were found to be lower in achalasia patients than in HVs. Thus, the findings indicate that the activity of the PI3K-AKT/mTOR pathway is low in achalasia.

Following the binding of a cytokine to a cell surface receptor in the JAK-STAT pathway, receptor dimerization occurs, followed by activation of JAK tyrosine kinases that are structurally associated with the receptor. Specific tyrosine residues on the receptor are phosphorylated by activated JAKs and form binding sites for a family of latent cytoplasmic transcription factors known as STATs. The STATs are phosphorylated by JAKs and then dimerize, leave the receptor, and migrate to the nucleus to enable the expression of pathway-associated genes.^26^ In this study, *STAT1, STAT2, STAT3, JAK*1, and *JAK2* gene expression levels were lower in the achalasia group than in the HV group. The SOCS family activated by cytokines is a negative regulator of the JAK-STAT pathway. The JAK triggers ubiquitination and proteasomal degradation of the protein.^9^ The *SOCS1* and *SOCS3* gene expression levels in achalasia patients were significantly increased compared to those in HVs. Protein levels of the phosphorylated form of STAT3 were also investigated, and no difference was found between the groups. Considering the gene expression results, it can be stated that the JAK-STAT pathway activity is lower in patients with achalasia than in HVs. In addition, the MAPK/ERK and PI3K/AKT pathways are involved in the downstream regulation of the JAK-STAT pathway.^27^^,^^28^ In the study, important findings were obtained showing that these 2 pathways are less active in achalasia than in HVs. This may provide evidence that the activity of the JAK-STAT pathway is also low in achalasia. The JAK-STAT pathway plays a critical role in immune responses and inflammation. Its downregulation could impair the ability of esophageal cells to respond to inflammatory stimuli or to mount an effective immune response. Given the possible autoimmune or inflammatory component in achalasia,^29^ reduced JAK-STAT signaling could be relevant to disease pathogenesis.

Another aim of this study was to determine the esophageal epithelial barrier function characteristics in achalasia patients. According to the results, the esophageal epithelium of patients with achalasia may not be predisposed to GERD after achalasia treatment because the permeability and resistance of the tissues were similar to those of tissues from healthy controls. To the best of knowledge, no studies have investigated TER and permeability of the esophageal epithelium in achalasia group. Studies using the mini-Ussing chamber system are generally based on the comparison of HVs with subtypes of GERD. It was found that the TEER and permeability values in achalasia patients were not significantly different from those in HVs, although they numerically reached a higher value in the achalasia group. Ates et al^30^ developed a minimally invasive mucosal impedance (MI) device to measure esophageal injury and included HV, achalasia, NERH, ERH, and eosinophilic esophagitis (EE) groups in their study. MI values were determined by touching the impedance catheter to different points of the esophagus during endoscopies. They concluded that the MI values in the achalasia group were similar to those in the HV group and were significantly higher than those in the NERH, ERH, and EE groups. Their findings in the achalasia epithelium using a different method gave results similar to those obtained in the study. According to the mini-Ussing chamber data, it was hypothesized that long-term exposure of the esophagus to lactic acidosis and retention of food and other substances in achalasia patients, which might lead to mechanical disruption damage, would not affect the esophageal epithelial permeability properties if not augmented.

In this study, the gene and protein expression levels of CDH1, ZO-1, ZO-2, CLDN1, CLDN4 and OCLN were investigated. These 6 molecules were selected for study based on their functions. OCLN is critical for the formation of tight junctions in most tissues.^31^ CLDN1 and CLDN4 are molecules that participate in high barrier function and close the intercellular space,^32^ and ZO-1 and ZO-2 are involved in the relationship between tight junctions on the epithelial surface and the cytosol.^33^ CDH1 protein bridges both surround the cell membrane and supports the union of OCLN and CLDN, and they have an integral role in establishing junction (electrical) resistance and controlling junction permeability.^34^ For these reasons, the molecules chosen constitute important components of the tight junction complex. The gene levels of molecules associated with epithelial barrier function were significantly high in achalasia patients, but only the ZO-2 protein level was consistent with the gene results. In a study by Zhu et al,^35^ TJP1 and CLDN1 protein levels were found to increase when miRNA‑29 was suppressed in irritable bowel syndrome patients. In another study, it was shown that miRNA-596 and miRNA-3620-3p play a role in reducing CLDN4 expression.^36^ miRNA studies in achalasia are limited in the literature, and there are no studies that have targeted tight junctions. The discrepancies between gene and protein expression levels observed in the study may result from a combination of posttranscriptional, translational, and post-translational regulatory mechanisms. These may include miRNA interference, mRNA instability, impaired translation efficiency, and enhanced protein degradation via the ubiquitin-proteasome system, as well as abnormalities in protein folding or trafficking.^37^ Further studies incorporating miRNA profiling or proteomic analysis would help elucidate the underlying mechanisms.

The study has some limitations. The number of patients with type III achalasia was small, reflecting the rarity of this subtype. Furthermore, due to the limited amount of biopsy tissue, it was not possible to assess protein expression for all the genes studied. Although mechanical parameters such as LES pressure are clinically relevant in achalasia, their evaluation was beyond the scope of this study, which focused specifically on epithelial molecular and electrophysiological properties. Nevertheless, the combination of transcriptomic, proteomic, and electrophysiological data provides a comprehensive picture of epithelial alterations in achalasia.

This study is the first to comprehensively evaluate the electrophysiological and molecular properties of the esophageal epithelium in achalasia. The findings indicate that major cell signaling pathways are markedly suppressed in the epithelial tissue of these patients.

Despite the absence of esophageal peristalsis, epithelial barrier function was preserved, with no significant alterations in tissue resistance or permeability. This suggests that the esophageal epithelium may not be inherently predisposed to reflux-related injury following therapeutic interventions such as POEM.

The molecular insights presented here may help direct future studies exploring similar signaling mechanisms in the muscular or neuronal compartments of the esophagus. Nevertheless, this study provides important groundwork for understanding epithelial integrity in achalasia and its potential clinical relevance in GERD risk management post-treatment.

## Figures and Tables

**Table 1.** Demographic Characteristics of Patients with Achalasia and Healthy Volunteers

Variable	Achalasia (n = 37)	HVs (n = 15)
Women, %	62	40
Age, mean (SD), years	42.8 (7.2)	40.5 (9.8)
BMI, mean, kg/m^2^	24.6	23.6

BMI, body mass index; HV, healthy volunteer; SD, standard deviation.

**Table 2.** Relative Gene Expression Levels of Epithelial Barrier Molecules in Achalasia and Healthy Volunteers

Gene	Fold Change	*P*
*CDH1 *	3.81	.002
*OCLN *	2.97	.045
*CLDN1 *	3.40	.023
*CLDN4 *	3.28	.046
*ZO-1 *	3.85	.001
*ZO-2 *	5.90	.003

Gene expression normalized to ACTB and B2M. Values represent fold change. *P*-values were determined using unpaired *t*-tests; *P* < .05 considered significant.

**Table 3.** Protein Levels of Epithelial Junctional Molecules Measured by ELISA. Only ZO-2 Protein Levels were Significantly Different Between Groups (*P = .001)

Protein (ng/mL)	Achalasia (n = 37)			Healthy Volunteer (n = 15)		
	Average (n = 37)	SD	Median	(Average n = 15)	SD	Median
CDH1	8.372	0.540	8.330	7.347	0.753	7.864
OCLN	18.538	5.850	20.147	14.260	10.639	20.983
CLDN1	1.711	1.753	1.208	1.781	0.712	1.968
CLDN4	2.515	0.857	2.362	2.216	1.468	1.377
ZO-1	9.018	0.882	9.127	8.056	0.821	7.963
**ZO-2**	3.191*	1.608	2.618	1.224	0.746	1.064

*P = .001 compared to HVs.

**Table 4.** Significantly Altered Cell Signaling-Related Gene Expressions in Achalasia Compared with HVs

Gene	Fold Change	*P*
*BCL2L1 *	31.72	.047
*IFNA1 *	25.82	.036
*IL10 *	20.58	.026
*IFNB1 *	17.72	.030
*NLRP12 *	16.87	.034
*IL6 *	13.05	.035
*IL2RA *	8.21	.044
*SOCS1 *	8.15	.006
*IL1R1 *	5.73	.012
*AGT *	4.89	.040
*RELB *	4.16	.033
*HTR2B *	3.76	.015
*AKT2 *	3.62	.038
*TICAM2 *	3.44	.042
*SOCS3 *	2.99	.016
*TNFAIP3 *	2.98	.041
*BIRC2 *	2.61	.015
*BCL2 *	2.49	.024
*GJA1 *	2.45	.001
*TNF *	2.42	.049
*MAPK1 *	−2.09	.002
*JAK2 *	−2.43	.001
*EGF *	−2.74	.019
*RAN *	−2.95	.049
*JAK1 *	−3.02	.030
*FOS *	−3.49	.006
*MAP2K1 *	−3.73	.042
*PSMA3 *	−3.73	.008
*NRAS *	−3.77	.005
*HRAS *	−4.01	.001
*STAT2 *	−4.31	.006
*STAT1 *	−5.72	.045

Normalization used ACTB, B2M, and PPIA. Genes listed showed *P* < .05. Positive values indicate upregulation; negative values indicate downregulation.

**Table 5.** Expression of Phosphorylated Cell Signaling Proteins in Achalasia and HVs

Protein (FI)	Achalasia (n = 37)			Healthy Volunteer (n = 15)		
	Average	SD	Median	Average	SD	Median
Akt (Ser473)	17.833*	0.885	18.000	20.028	1.826	19.750
c-Jun (Ser63)	32.833*	7.112	31.000	50.917	11.587	50.500
Erk1/2 (Th202/Tyr204)	33.071*	3.392	32.500	60.361	24.8515	48.750
IκB-α (Ser32/Ser36)	20.571*	2.404	21.000	88.111	60.326	76.500
MEK1 (Ser217/Ser221)	13.286*	2.028	13.000	159.972	54.266	102.500
mTOR (Ser2448)	24.429*	3.381	23.000	96.500	77.562	72.500
Stat3 (Tyr705)	67.357	3.616	67.000	66.833	3.658	67.000

Measured by multiplex ELISA. Proteins with *P* < .05 are marked with an asterisk (*). Data presented as fluorescence intensity (FI), SD, and median.

*P < .05 compared to HVs.

## Data Availability

The data that support the findings of this study are not publicly available due to their containing information that could compromise the privacy of research participants. However, the data are available from the corresponding author, S.K. (kipcaksezgi@gmail.com), upon reasonable request.
